# Synaptic Vesicle Recycling at the Developing Presynapse

**DOI:** 10.1111/jnc.70206

**Published:** 2025-08-27

**Authors:** Nawon Kim, Michael A. Cousin

**Affiliations:** ^1^ Centre for Discovery Brain Sciences University of Edinburgh Edinburgh UK; ^2^ Muir Maxwell Epilepsy Centre University of Edinburgh Edinburgh UK; ^3^ Simons Initiative for the Developing Brain University of Edinburgh Edinburgh UK

**Keywords:** calcium, endocytosis, exocytosis, neurotransmitter, presynapse, synaptic maturation, synaptogenesis, vesicle

## Abstract

Neurotransmitter release plays a fundamental role in brain communication. This is mediated via the exocytosis of neurotransmitter‐containing synaptic vesicles (SVs) at the presynapse. After fusion with the presynaptic plasma membrane, SVs are regenerated by endocytosis and recycled back into functional pools. The great majority of research that has studied this essential process has focused on SV recycling at the mature presynaptic terminal. In contrast, SV recycling in immature neurons remains poorly understood, even though its disruption is heavily associated with a series of neurodevelopmental disorders. Evidence is accumulating that developing neurons display distinct presynaptic mechanisms for SV recycling. For example, developing presynapses display loose coupling between evoked calcium influx and SV fusion, with spontaneous SV exocytosis and clathrin‐mediated endocytosis being the dominant exocytosis and endocytosis mechanisms respectively. In contrast, SV fusion in mature nerve terminals is tightly coupled to evoked calcium influx, with evoked SV exocytosis and endosomal modes of endocytosis dominant. This article reviews research at each stage of the SV recycling process at both mature and immature nerve terminals, beginning with the coupling of activity‐dependent calcium influx to neurotransmitter release. In doing so, it aims to provide an integrated perspective of current knowledge regarding SV recycling through development across different neuronal systems, while posing key future questions to address for the presynaptic development field.

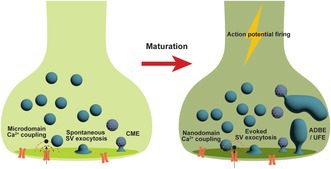

## Graphical abstract

During presynaptic development, the coupling between calcium influx and synaptic vesicles (SVs) tightens with maturity, transitioning from microdomain to nanodomain. In immature neurons, spontaneous SV exocytosis is the dominant SV fusion mode, whereas SV fusion is mostly evoked by action potential firing in mature presynapses. Finally, SVs are recycled through clathrin‐mediated endocytosis (CME) at immature presynapses, which is mostly replaced by clathrin‐independent pathways (activity‐dependent bulk endocytosis [ADBE] and ultrafast endocytosis [UFE]) throughout development.

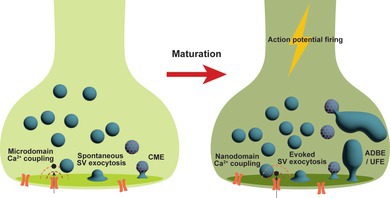

**Abbreviations**
ωAgTx‐IVA: ω‐agatoxin‐IVA
ωCgTx‐GVIA: ω‐conotoxin‐GVIA
ADBE: activity‐dependent bulk endocytosis
AP2: adaptor protein complex 2
APV: 2‐amino‐5‐phosphonovaleric acid
BAPTA: 1,2‐bis‐(2‐aminophenoxy)ethane‐*N*,*N*,*N*′,*N*′‐tetraacetic acid
[Ca^2+^]_i_
: intracellular free calcium
CaN: calcineurin
CME: clathrin‐mediated endocytosis
CNQX: cyanquixaline
DIV: days in vitro
EGTA: ethylene glycol‐bis(2‐aminoethylether)‐*N*,*N*,*N*′,*N*′‐tetraacetic acid
EPSC: excitatory postsynaptic current
GABA: gamma‐aminobutyric acid
IPSC: inhibitory postsynaptic current
mEPSC: miniature EPSC
mIPSC: miniature IPSC
NMDA: *N*‐methyl‐d‐aspartate
NSF: *N*‐ethylmaleimide‐sensitive fusion
RRP: readily releasable pool
SDS‐FRL: SDS‐digested freeze‐fracture replica labelling
sEPSC: spontaneous EPSC
sIPSC: spontaneous IPSC
SNA: spontaneous network activity
SNAP25: synaptosomal‐associated protein of 25 kDa
SNARE: NSF attachment protein receptor
SV: synaptic vesicle
Syt1: synaptotagmin‐1
TTx: tetrodotoxin
UFE: ultrafast endocytosis
VGCC: voltage‐gated calcium channel

## 1 Introduction

A human brain consists of billions of neurons, which form over a hundred trillion connection points called synapses (Herculano‐Houzel 2009). These synapses convey information from one neuron to another by releasing neurotransmitters from the presynapse to the postsynapse. Synaptic density reaches its maximum in the first few years of life, which constitute a critical period for brain development (Huttenlocher 1999). The connections that are established during this defined time window form the basis for the majority of information storage and behaviour in later life. Central to the optimal functioning of these brain circuits is the fidelity of neurotransmitter release, which is triggered by precise patterns of neuronal activity.

The process of neurotransmitter release is constrained in both time and space at the presynapse, with a discrete series of molecular stages tightly coupled to activity‐dependent calcium influx. This influx of calcium is spatially regulated, with a high concentration of voltage‐gated calcium channels (VGCCs) anchored at the active zone, which is the site of neurotransmitter release (Gundelfinger and Fejtova 2012; Simms and Zamponi 2014). The active zone contains a series of scaffolding molecules that localise calcium channels in close proximity to the release machinery (Gundelfinger et al. 2016). The spatial organisation results in the creation of a transient microdomain of extremely high intracellular free calcium ([Ca^2+^]_i_) (Neher and Sakaba 2008), which is sensed by the calcium trigger for synchronous neurotransmitter release, synaptotagmin‐1 (Syt1) that binds calcium with extremely low affinity (Fernández‐Chacón et al. 2001; Geppert et al. 1994; Nishiki and Augustine 2004). The formation of the transient calcium microdomain at the active zone, in addition to the low affinity of Syt1 for calcium, ensures that neurotransmitter release is exclusively reserved to this defined presynaptic structure.

Chemical neurotransmitters are stored inside synaptic vesicles (SVs), which are small and clear lipid‐bilayer structures of approximately 40 nm in diameter. Neurotransmitter is loaded into SVs against its concentration gradient by exploiting a protonmotive force generated by a V‐type ATPase located on the SV membrane (Gowrisankaran and Milosevic 2020; Pulido and Ryan 2021). Neurotransmitter release is mediated by three key molecules, collectively termed soluble *N*‐ethylmaleimide‐sensitive fusion (NSF) attachment protein receptor (SNARE) proteins, which reside on SV or presynaptic plasma membranes respectively (Jahn et al. 2024). Synaptobrevin‐2 is located on SVs, whereas syntaxin‐1 and synaptosomal‐associated protein of 25 kDa (SNAP25) are concentrated on the plasma membrane. All the SNARE proteins contain motifs that facilitate a processive association (colloquially termed ‘zippering’) that drives the fusion of the SV with the plasma membrane (Rizo et al. 2024; Weber et al. 1998).

This fusion event is preceded by docking and priming. Docking is the process of the physical attachment of SVs to the plasma membrane of the release site, while priming renders SVs fusion‐competent by changing the conformation of the SNARE complex, a process coordinated via interactions with a series of molecules including Munc‐13, Munc‐18, complexin and Syt1 (An and Lindau 2024; Imig et al. 2014; Rizo 2022). On depolarisation of the presynapse during action potential invasion, docked and primed SVs fuse with the plasma membrane to release their neurotransmitter content into the synaptic cleft. As mentioned earlier, this is triggered by the binding of free calcium ions to Syt1, which drives altered interactions with both the assembled SNARE complex and plasma membrane, resulting in the synchronisation of neurotransmitter release to neuronal activity (Hui et al. 2009; Toulmé et al. 2024).

**FIGURE 1:**
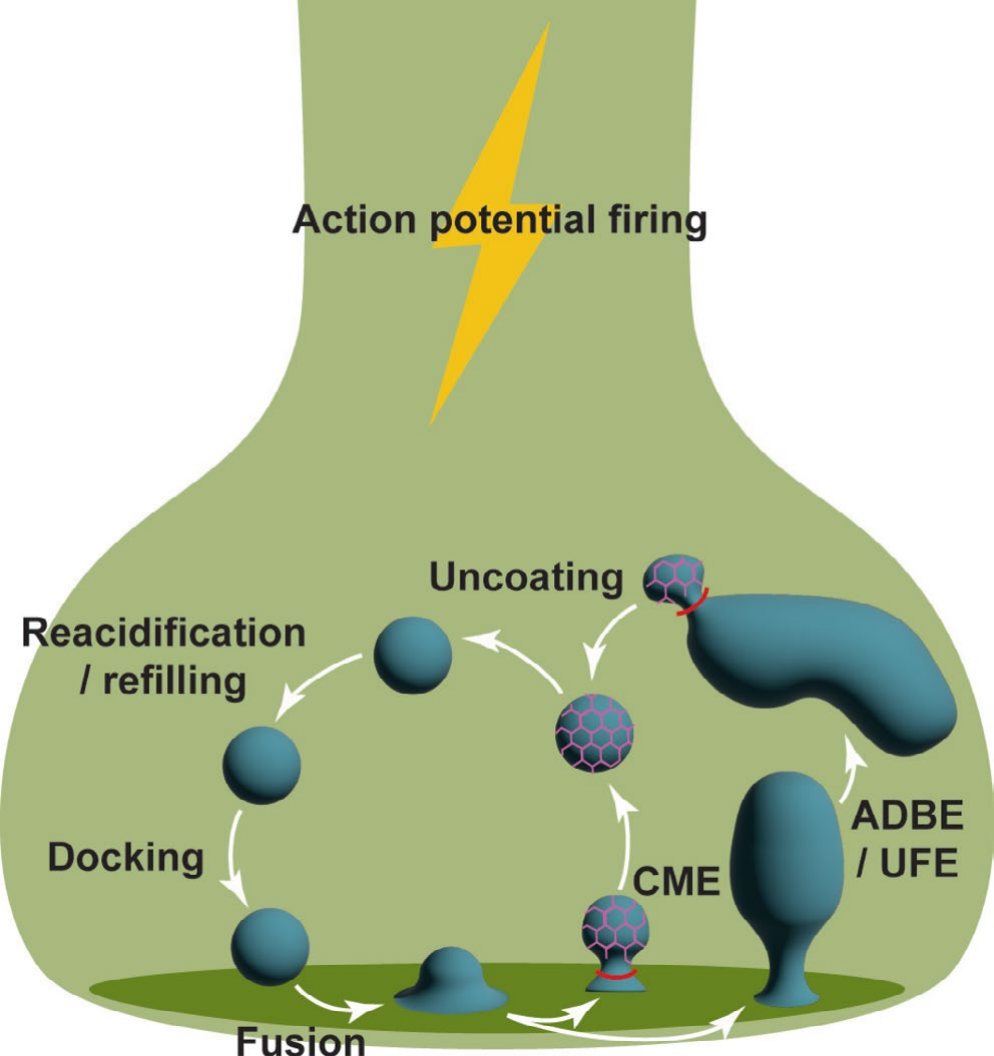
SV recycling pathways. SVs are docked to the active zone and fuse with the plasma membrane on calcium influx. SV cargoes and membrane are then retrieved via endocytosis. These include clathrin‐mediated endocytosis (CME), activity‐dependent bulk endocytosis and ultrafast endocytosis (UFE) (Chanaday et al. 2019). In CME, clathrin‐coated pits (pink) invaginate from the plasma membrane with the neck of the invaginated pit constricted by a dynamin ring (red) to produce clathrin‐coated vesicles. In ADBE/UFE, endosomes are generated from the plasma membrane in a dynamin‐dependent, but clathrin‐independent manner. SV production from these endosomes is both clathrin‐ and dynamin‐dependent. In all cases, regenerated SVs are uncoated, acidified and filled with neurotransmitters to replenish SV pools.

Following neurotransmitter release, SV membrane and proteins are deposited on the plasma membrane. The SNARE complex is still assembled at this stage and will be in the cis‐configuration. This cis‐SNARE complex is disrupted by the action of the ATPase NSF, which is recruited via soluble NSF attachment proteins (Yoon and Munson 2018). NSF‐dependent ATP hydrolysis frees syntaxin‐1 and SNAP‐25 to enter new trans‐SNARE complexes on the plasma membrane, with synaptobrevin‐2 liberated for its retrieval via endocytosis. Numerous studies suggest that synaptobrevin‐2 and other SV cargo proteins are clustered at the periphery of the active zone for subsequent retrieval by endocytosis (Cousin 2017; Gimber et al. 2015). There are multiple endocytosis modes that regenerate SVs from the presynaptic plasma membrane which are proposed to be selected dependent on the intensity of previous neuronal activity. These pathways are clathrin‐mediated endocytosis (CME), ultrafast endocytosis (UFE) and activity‐dependent bulk endocytosis (Figure 1; Chanaday et al. 2019).

CME is an established mechanism of cargo retrieval in all mammalian cells and was originally proposed to be the dominant mode of SV retrieval at the presynapse (Granseth et al. 2006). A key aspect of CME is the clustering of SV cargo proteins by adaptor protein complex 2 (AP2), which both identifies cargo for retrieval and the recruitment of subsequent clathrin coat proteins (Kelly and Owen 2011; McMahon and Boucrot 2011; Robinson 2004). This results in the direct generation of individual SVs from the plasma membrane by invagination and then fission of clathrin‐coated SVs mediated by the GTPase dynamin‐1 (Antonny et al. 2016). However, the dominant role of CME has been questioned by more recent experiments. The Watanabe group showed the clathrin‐independence of invagination of the plasma membrane by exploiting technology that allowed the visualisation of endocytic intermediates just 50 ms after stimulation (Watanabe, Liu, et al. 2013; Watanabe, Rost, et al. 2013; Watanabe et al. 2014). This mode of endocytosis was identified as UFE and formed small endosomes directly from the plasma membrane.

Another clathrin‐independent endocytosis mode is ADBE. Similar to UFE, it has a two‐step mechanism involving initial endosome formation followed by generation of individual SVs from the endosomal structures (Clayton and Cousin 2009). Central to this mechanism is the coordinated dephosphorylation of a subset of endocytosis proteins called the dephosphins, by the calcium‐dependent protein phosphatase calcineurin (Clayton and Cousin 2009; Cousin and Robinson 2001; Imoto et al. 2022, 2024). ADBE was initially proposed to be an effective way to rapidly retrieve a large area of plasma membrane to maintain membrane tension. Individual SVs are generated from the bulk endosome through similar cargo recognition mechanisms as CME (clathrin and adaptor proteins) (Cheung and Cousin 2012; Ivanova et al. 2021; Kononenko et al. 2014), but the endosome also serves as a sorting platform to recycle or degrade cargo proteins via the endolysosomal system (Ivanova and Cousin 2022).

Evidence is accumulating that these different modes of SV endocytosis either share the same mechanism or are closely integrated. For example, depletion of clathrin heavy chain arrests SV generation from endosomes generated via UFE (Watanabe et al. 2014), in an identical manner to ADBE (Ivanova et al. 2021; Kononenko et al. 2014). UFE and ADBE may also share the same molecular mechanism, although they were originally proposed to be triggered by mild and intense stimulation respectively (Blumrich et al. 2023; Clayton and Cousin 2009; Imoto et al. 2022, 2024; Smillie et al. 2005; Watanabe et al. 2018; Xue et al. 2011). Intriguingly, some atypical SNARE and Syt proteins, such as VAMP4 and Syt7, may bias the triggering of different endocytosis modes in an indirect manner via their control of asynchronous release (Li et al. 2017; Lin et al. 2020; Raingo et al. 2012). It is highly likely that CME, UFE and ADBE are part of one convergent endocytosis mode, with the slower CME initiating on the plasma membrane but completing on endosomes formed via UFE/ADBE.

SV recycling takes place at the presynapse, which is specified by the localisation, organisation and activation of various ion channels, active zone scaffolding proteins and exo‐/endocytic proteins. Synaptogenesis starts as early as five gestational weeks in humans and peaks during the first two postnatal years (Budday et al. 2015). Rodents display a much earlier peak of synaptogenesis, which is typically maximal at the second to third postnatal week, although some recent publications report steady synaptogenesis until young adulthood (Semple et al. 2013; Wildenberg et al. 2023). Because of this, acute brain slices from rodents are typically thought of as mature after at least 1 month. Neuronal rodent cultures are usually deemed mature after approximately 2 weeks in culture, on cessation of synaptogenesis and establishment of bona fide synapses. In more detail, processes start to develop in culture within 2 days after plating, but axonal identity is established with the localisation of the axon initial segment starting from around 5 days in vitro (DIV) (Bolz and Haucke 2024; Dotti et al. 1988; Yang et al. 2007). Minor processes continue to develop in parallel to axons, which specify into dendrites. This process of synaptogenesis is then followed by a stationary phase, during which synapses mature (Kim et al. 2024). The overwhelming majority of our current knowledge of SV recycling mechanisms, which is summarised above, originates from studies of mature neurons, either in neuronal culture or acute brain slices. However, whether these nascent synapses possess the same SV recycling events and regulation is unknown. This gap of knowledge is striking, since it is highly likely that immature neurons operate not just as simplified versions of mature neurons, but instead may have distinctive SV exocytosis and endocytosis processes, which may be essential for the establishment and regulation of neuronal circuits (Andreae and Burrone 2015; Cho et al. 2015; Choi et al. 2014; Kim et al. 2024).

This review will collate current knowledge of SV recycling in the context of presynaptic development, sequentially discussing calcium influx, SV fusion by exocytosis and finally SV reformation by endocytosis. Since this is a vast topic that spans neuronal differentiation, neuronal migration, axon specification and synaptogenesis, the scope of this review will be focused on SV recycling at the developing presynapse. Therefore, events that control the formation of the presynaptic precursor—growth cones, transport and assembly of presynaptic molecules and maintenance of axonal polarity via the axon initial segment will not be covered (Bolz and Haucke 2024). For more extensive coverage of these specific topics, refer to other reviews (Leterrier 2018; Nozumi and Igarashi 2018; Petzoldt 2023; Rizalar et al. 2021).

## 2 Coupling of Calcium Influx to SV Fusion

At the mature presynapse, influx of calcium ions through VGCCs triggers fusion of SVs at the active zone via calcium binding of Syt1 (Fernández‐Chacón et al. 2001; Geppert et al. 1994; Nishiki and Augustine 2004). Syt1 is expressed robustly and specifically at presynaptic compartments as early as three DIV in cultured neurons, so much so that its presence was assumed to mark potential presynaptic sites or precursor packets for active zone assembly (Kraszewski et al. 1995; Matteoli et al. 1992). However, recent findings suggest that the spatial organisation of VGCCs and the topography of SV fusion are different in developing neurons, indicating that there could be varying degrees of calcium coupling during presynaptic development.

VGCCs can be categorised into five subtypes in neurons: L‐ (Ca_v_1.1–1.4), P/Q‐ (Ca_v_2.1), N‐ (Ca_v_2.2), R‐ (Ca_v_2.3) and T‐ (Ca_v_3) type (Simms and Zamponi 2014). In mature neurons, P/Q‐ and N‐type VGCCs account for the majority of the evoked calcium influx in both excitatory and inhibitory mammalian presynapses, which was revealed by exploiting pharmacological blockers for VGCC subtypes or by knocking out a specific VGCC subunit (Dolphin and Lee 2020; Reid et al. 2003). Similarly, the composition of VGCC subtypes that are coupled to neurotransmitter release at the nascent presynapse has been interrogated in numerous studies via the use of toxins that selectively inhibit specific subtypes. These studies indicate that the subtypes coupled to SV fusion appear to change during the transition to presynaptic maturity. For example, inhibitory neurotransmission at both cerebellar and thalamic synapses and excitatory neurotransmission in the brainstem displayed decreasing sensitivity to the cone snail toxin ω‐conotoxin‐GVIA (ωCgTx‐GVIA, which blocks N‐type VGCCs) in the second postnatal week in rat brain slices (Iwasaki et al. 2000; Iwasaki and Takahashi 1998). In agreement, imaging of [Ca^2+^]_i_ levels in cortical brain slices using the dye Fluo‐5F revealed a decrease in ωCgTx‐GVIA sensitivity in layer V pyramidal neurons from P8–10 to P21–24 (Bornschein et al. 2019). It was suggested that the rearrangement of VGCC localisation at the active zone was the driving force behind reduced ωCgTx‐GVIA sensitivity at maturing nerve terminals (Pravettoni et al. 2000). In this work, the contribution of N‐type VGCCs to the somatic calcium current increased through development, with a concomitant decrease in the sensitivity to ωCgTx‐GVIA of excitatory neurotransmitter release in autaptic cultures from DIV 3–4 to DIV 10–12. However, the decrease in the contribution of N‐type VGCCs to presynaptic calcium influx may vary depending on the type of synapse, as glycinergic inhibitory synapses in the spinal dorsal horn retain sensitivity to ωCgTx‐GVIA to maturity, as do non‐NMDA (*N*‐methyl‐d‐aspartate) excitatory synapses in cerebral cortical neurons according to whole‐cell recordings of evoked excitatory postsynaptic currents (EPSCs) and inhibitory postsynaptic currents (IPSCs) (Iwasaki et al. 2000). In contrast, calcium influx at some presynapses has little contribution from N‐type VGCCs throughout their development. For example, the gamma‐aminobutyric acid (GABA)ergic (Briševac et al. 2021) Basket cell‐Purkinje cell synapse in the cerebellar cortex presents a predominance of P/Q‐type VGCCs throughout their development from P7–9 to P21–23, with minimal ωCgTx‐GVIA sensitivity observed when the amplitude of evoked IPSCs was examined (Chen et al. 2024).

Time‐course analyses using different VGCC blockers have provided detailed information of subtype composition that contributes to both presynaptic calcium influx and coupling to neurotransmitter release. However, this is still a relatively crude measure since the high synchronicity of neurotransmitter release is reliant on the efficiency of coupling of calcium influx to SV fusion. Intriguingly, an increasing tightness of neurotransmitter release to calcium influx is one of the key hallmarks of presynaptic maturation, suggesting physical rearrangement of either or both VGCCs and the fusion machinery occurs at the active zone. The Calyx of Held in the medial nucleus of the trapezoid body is routinely the model of choice to monitor developmental changes in calcium coupling due to its accessibility for patch clamp capacitance methods. The ability to patch clamp this atypical nerve terminal facilitates delivery of small molecules directly to the presynapse and permits direct measurements of both calcium influx and SV fusion (via membrane capacitance) (Neher 2017).

Many studies examining calcium coupling to SV fusion have exploited the calcium buffers ethylene glycol‐bis(2‐aminoethylether)‐*N*,*N*,*N*′,*N*′‐tetraacetic acid (EGTA) and 1,2‐bis‐(2‐aminophenoxy)ethane‐*N*,*N*,*N*′,*N*′‐tetraacetic acid (BAPTA). Both buffers have near identical *K*_d_ for calcium of approximately 500 nM; whereas, EGTA binds calcium much more slowly (70 μs compared to 2 μs for BAPTA) (Adler et al. 1991). Therefore, the competition between EGTA and Syt1 for access to calcium influx can reveal important information about the physical association between VGCCs and the SV fusion machinery. At the Calyx of Held, EPSC amplitude in response to axonal stimulation was decreased by EGTA before hearing onset at P8–12 but not at P16–18, when hearing begins (Fedchyshyn and Wang 2005). In contrast, BAPTA was equally efficient in inhibiting EPSCs in both age groups (Fedchyshyn and Wang 2005). Based on these electrophysiological observations, the authors suggested that a transition takes place at the second postnatal week, from microdomain calcium coupling (loose) to nanodomain calcium coupling (tight). Two later studies examined whether this was due to a change in the spatial topography of the active zone or an intrinsic change in the release machinery using patch clamp electrophysiology, calcium uncaging and calcium dye imaging (Kochubey et al. 2009; Wang et al. 2008). First, when evoked EPSC amplitude and presynaptic calcium charge were correlated, the same amount of calcium influx resulted in increased glutamate release at P12–15 synapses compared to those from P7–10 (Kochubey et al. 2009). A combined assay of EPSC recording with calcium dye imaging following calcium uncaging confirmed a tightening between calcium influx and SV fusion between P9–11 and P16–19 (Wang et al. 2008). Intriguingly, when experiments were performed at physiological levels of extracellular calcium, there appeared to be a slight decrease in the efficiency of coupling, which was proposed to be due to an overall decrease in the average number of VGCCs controlling the fusion of a single SV (Fedchyshyn and Wang 2005; Wang et al. 2008). The spatial tightening of the SV fusion machinery to calcium influx at the Calyx of Held was further confirmed with electron microscopy (Nakamura et al. 2015). In this work, the authors complemented a similar electrophysiological approach to that described above with an SDS‐digested freeze‐fracture replica labelling (SDS‐FRL) technique, which was used to report the size of Ca_v_2.1 clusters and the number of Ca_v_2.1 channels in each cluster, both of which increased in the second postnatal week. The authors hypothesised that the VGCC clusters regulate the fusion of SVs within a certain distance, which was simulated in silico (Nakamura et al. 2015). Therefore, multiple studies have demonstrated increased coupling of SV fusion to calcium influx with ongoing presynaptic maturity, which appears to be dictated by the physical distance between the release machinery and VGCCs.

The Calyx of Held is an atypical synapse because of its giant size and its high number of active zones (Baydyuk et al. 2016). To test whether the developmental tightening of calcium coupling to neurotransmitter release can be extrapolated to typical small synapses, evoked EPSCs at glutamatergic parallel fibre–Purkinje cell synapses were investigated in cerebellar slices (Baur et al. 2015). These synapses also displayed decreased sensitivity to EGTA from P8–10 to P21–24, suggesting the increase in coupling is a universal consequence of presynaptic maturity. Furthermore, using the same SDS‐FRL technique as Nakamura et al. (2015), the authors showed that Ca_v_2.1 particles were arranged closer together at the active zone in mature neurons, strongly supporting that the mechanism behind tighter calcium coupling is the spatial reorganisation of VGCCs within the active zone (Baur et al. 2015). These findings were corroborated by studies that recorded evoked EPSCs in pyramidal neurons from mouse neocortical slices (Bornschein et al. 2019). The same strategies of calcium chelation using EGTA and BAPTA were employed to measure calcium coupling, with calcium dye used to report presynaptic calcium transients. Once again, evoked EPSCs showed reduced EGTA sensitivity in mature slices (P21–24) when compared to immature equivalents (P8–10). This increased coupling occurred coincident with an increasing contribution from P/Q‐type VGCCs (Bornschein et al. 2019). This suggests that the VGCC rearrangement at the active zone that results in tightening of calcium coupling to SV fusion may also involve replacement of specific VGCC subtypes. The increased coupling through maturity is not exclusive to excitatory synapses, since inhibitory neurotransmission from Basket cells to Purkinje cells in cerebellar slices also displayed reduced sensitivity to EGTA from measurements at P7–9 to those at P14–16 (Chen et al. 2024). Finally, studies that examined the distance between Ca_v_2.1 channels and the active zone protein Munc13 using the super‐resolution microscopy technique 3D‐STORM revealed a reduction in separation from P6 to 18 in lemniscal fibres in the ventral posterior medial nucleus (Midorikawa et al. 2024). Therefore, there appears to be a growing consensus that exocytosis becomes more tightly coupled to the calcium influx due to the closer physical organisation of VGCCs to the SV fusion machinery, with varying degrees depending on the types of synapses (Rebola et al. 2019). This should result in both higher fidelity and synchronicity of neurotransmission during the progression to presynaptic maturity.

## 3 SV Exocytosis

The increasing fidelity and synchronicity of action potential‐driven neurotransmission during development is a requirement to ensure accurate communication across complex circuits. However, SV fusion events can also occur in the absence of neuronal activity. For the purposes of this review, we define these events as spontaneous exocytosis, which is action potential‐independent but calcium‐dependent fusion of SVs, while acknowledging evidence for calcium‐independent spontaneous SV fusion events, which are observed frequently in developing neurons (Glitsch 2008; Liu et al. 2018; Truckenbrodt and Rizzoli 2014; Williams and Smith 2018). A significant amount of work has investigated the properties and mechanism of spontaneous exocytosis, which is summarised below. How evoked neurotransmitter release changes through development will be reviewed in the following section.

### 3.1 Spontaneous Exocytosis

At the mature synapse, spontaneous release is evoked by calcium influx, but whether the calcium influx is generated by VGCCs or other sources remains controversial, especially in glutamatergic synapses (Babiec and O'Dell 2018; Chanaday et al. 2021; Dai et al. 2015; Liu et al. 2018; Reese and Kavalali 2015; Williams and Smith 2018; Xu et al. 2009). There is more consensus surrounding the VGCC‐dependency of miniature IPSCs (mIPSCs), primarily due to studies that revealed a decrease in mIPSC frequency in cortical and hippocampal cultured neurons when recorded in the presence of VGCC blockers such as ωCgTx‐GVIA and ω‐Agatoxin‐IVA (ωAgTx‐IVA, which inhibits P/Q‐type VGCCs) (Goswami et al. 2012; Williams et al. 2012). There is also growing evidence that the calcium required for miniature EPSCs (mEPSCs) is derived from VGCCs (Ermolyuk et al. 2013; Lee et al. 2022). In these studies, the reduction in mEPSC frequency in primary hippocampal neurons in the presence of VGCC blockers was reproduced in silico, with the assumption that VGCC opening occurs stochastically (Ermolyuk et al. 2013). The VGCC‐dependence of miniature events in hippocampal neurons was supported by studies in both hippocampal autaptic cultures and at the Calyx of Held after hearing onset (P18) (Lee et al. 2022). Intriguingly, this VGCC‐dependence was absent at the immature Calyx of Held (P9) (Lee et al. 2022), suggesting that spontaneous SV release could be subject to the same development‐dependent tightening of calcium coupling discussed above.

The calcium sensor for spontaneous SV exocytosis appears to be distinct from that required for action potential‐evoked neurotransmitter release. For example, Syt1 knockout neurons display a large increase in spontaneous fusion, concurrent with an almost complete ablation of evoked neurotransmitter release (Geppert et al. 1994). A leading candidate is Doc2, which is a cytoplasmic C2 domain‐containing protein that has affinity for calcium at least two orders of magnitude higher than Syt1 (Groffen et al. 2010). Importantly, deletion of both *Doc2a* and *Doc2b* genes resulted in a profound inhibition of spontaneous fusion in neuronal culture, but little impact on evoked neurotransmitter release (Groffen et al. 2010). The calcium dependency of this control has been debated (Pang et al. 2011); however, it was later explained due to gain of function mutations within the calcium binding motif in molecular replacement studies (Courtney et al. 2018). This delineation of roles may be too simplistic; however, since the contribution of Syt1 and Doc2 to spontaneous release differs between excitatory and inhibitory neurons (Courtney et al. 2018).

Whereas the majority of the publications mentioned above explore spontaneous SV exocytosis in mature synapses, the process is highly prevalent in developing neurons. In fact, SVs appear to possess an intrinsic capability to be recycled upon calcium influx even before postsynaptic contacts are established. For example, via the use of antibodies targeting the intra‐vesicular domain of Syt1, multiple groups have demonstrated that spontaneous exo‐/endocytosis occurs before postsynaptic contact, in some cases as early as DIV 3 in primary neuronal culture (Coco et al. 2002; Kraszewski et al. 1995; Matteoli et al. 1992). This event was confirmed to be dependent on calcium influx, since unloading of the amphiphilic dye FM1‐43 (which intercalates into the plasma membrane and becomes fluorescent) was dependent on calcium in 2‐day‐old *Xenopus* spinal cord neurons, and the internalisation of Syt1 antibodies was also dependent on the extracellular calcium concentration at DIV 2–4 in hippocampal neurons (Coco et al. 2002; Dai and Peng 1996). The dominance of spontaneous SV fusion events at immature synapses was confirmed in studies using biotin conjugated to the lumenal domain of synaptobrevin‐2 (biosyn) and impermeant fluorescent streptavidin in primary cultures of hippocampal neurons (Andreae et al. 2012). In these studies, the intensity of the biosyn signal (which was proportional to the number of recycling SVs) peaked at DIV 4 when streptavidin was applied for 15 min in the absence of neuronal activity, and then decreased to a third of this maximum by the second week in vitro (Andreae et al. 2012). It is assumed that the vesicles mediating these events are equivalent to typical SVs; however, this may not be the case. An alternative hypothesis is that the high prevalence of spontaneous events is due to fusion of ‘constitutively releasing vesicles’, which deliver key molecules to the developing synapse and after multiple fusion events acquire a typical SV identity (Truckenbrodt and Rizzoli 2014). However, it should be noted that the events reported to be due to constitutively releasing vesicles are calcium‐independent in nature.

Multiple groups have examined the role of spontaneous SV release in synaptic maturation. For example, it appears to contribute to presynaptic development in *Drosophila* (Cho et al. 2015; Choi et al. 2014). The earlier study used a series of genetic lines to selectively eliminate either evoked or spontaneous release or both (Choi et al. 2014). Elimination of vesicular glutamate transporter via a number of different genetic manipulations ablated both evoked and spontaneous release but had no impact on the number of total nerve terminals formed. Intriguingly, there was a large increase in the number of small boutons, suggesting synaptic transmission is required for subsequent nerve terminal growth (Choi et al. 2014). The selective elimination of evoked neurotransmitter release by either expression of tetanus toxin light chain (to cleave neuronal(n)‐synaptobrevin) or membrane‐tethered Plectreurys toxin II (which blocks cacophony, a *Drosophila* N‐type VGCC that regulates evoked but not spontaneous release) had no impact on bouton number or size. This striking result revealed that evoked SV exocytosis had no overt impact on presynaptic development, even when this parameter was enhanced by increased opening of voltage‐gated sodium channels (Choi et al. 2014). These results were corroborated in a different study where spontaneous release was enhanced via either knock‐down of the priming molecule complexin or overexpression of its phospho‐mimetic form (S126D) (Cho et al. 2015). These mutant lines reported an increase in the number and growth of nerve terminals in the same area of muscle, confirming that spontaneous fusion events are required for optimal bouton development (Cho et al. 2015).

The impact of spontaneous SV exocytosis is not limited to the presynapse. For example, it appears to be required for the clustering of postsynaptic receptors (Saitoe et al. 2001). This was revealed by comparing different *Drosophila* lines that were deficient in either evoked exocytosis (*syntaxin‐1A*, *n‐synaptobrevin*) or both evoked and spontaneous exocytosis (*cysteine string protein*, *shibire*). Importantly, glutamate receptor clustering at the larval NMJs of *Drosophila* was only disrupted in the latter two mutants but not the former ones (Saitoe et al. 2001). However, this result contrasts with initial studies in the Munc‐18 knockout mouse, which showed intact initial synaptogenesis despite the absence of neurotransmission (Verhage et al. 2000).

More recently, it was shown that spontaneous SV exocytosis functions as a long‐range signal that promotes dendritic complexity (Andreae and Burrone 2015). The authors confirmed that the glutamate released via spontaneous SV exocytosis is detected from the postsynapse, the majority of which was blocked by the NMDA receptor blocker 2‐amino‐5‐phosphonovaleric acid (APV). The postsynaptic response was reported by local calcium transients at the dendrites using the genetically encoded reporter GCaMP3, and it was reproduced when spontaneous SV exocytosis was mimicked by locally applying glutamate to the dendrites. To demonstrate the effects of spontaneous SV exocytosis at the postsynapse, the authors monitored dendritic growth during treatment with APV and cyanquixaline (CNQX) to block ionotropic glutamate receptors, or tetrodotoxin (TTx) to inhibit sodium channels and therefore activity, at early (DIV 1–3) and late (DIV 6–8) stages of neuronal development. The early treatment of APV and CNQX resulted in a decrease in dendrite length and in dendritic branching, which was distinct from the TTx‐treated group (Andreae and Burrone 2015).

### 3.2 Evoked SV Exocytosis

Compared to spontaneous SV release, whose developmental roles have been widely researched, there are fewer studies on the developmental trajectory of action potential‐driven evoked SV exocytosis, especially downstream of calcium influx. There are conflicting reports on whether evoked release in developing neurons is different from that in mature neurons, and if it is distinct from the calcium coupling discussed above. In the following paragraphs, we summarise the current literature in vitro, ex vivo and in vivo on evoked SV exocytosis in developing neurons.

In primary neurons from dissociated hippocampal culture, the transition in SV recycling mechanisms from spontaneous to evoked was highlighted in studies where SV turnover was revealed by biosyn labelling (Andreae et al. 2012). In these studies, neurons displayed negligible labelling in response to 60 mM KCl stimulation in the first week in vitro; however, evoked uptake was dominant from the second week. This confirmed earlier studies that monitored SV recycling using antibodies directed against the lumenal domain of Syt1 (Matteoli et al. 1992). These experiments revealed that almost all KCl‐evoked SV turnover at DIV 8 was inhibited by either removing extracellular calcium or after incubation with clostridial neurotoxins (Kraszewski et al. 1995; Coco et al. 2002). A more recent study in dissociated cultures of hippocampal neurons examined both the triggering and extent of evoked SV exocytosis using the genetically encoded reporter synaptophysin‐pHluorin (Kim et al. 2024). The work revealed that the number of nerve terminals that responded to action potential stimulation increased significantly from DIV 3 to DIV 14, in agreement with previous studies. However, there was no obvious increase in the extent of the synaptophysin‐pHluorin response at these boutons throughout development, suggesting that once nerve terminals become competent for evoked neurotransmitter release, they can operate close to optimal functionality (Kim et al. 2024). In contrast, the fraction of SVs undergoing action potential‐evoked fusion at individual nerve terminals dramatically increased between DIV 5–7 and DIV 14–28 in organotypic hippocampal slices (Rose et al. 2013). Using a ratiometric version of synaptophysin‐pHluorin, it was demonstrated that eventually all SVs fused in response to stimulation, in contrast to immature nerve terminals which only fused approximately 50%. Based on the findings that the presynapses in organotypic slices mature to mobilise all of their SVs, but the presynapses in dissociated cultures do not recapitulate the change, the authors raise potentially profound questions regarding the applicability of cultured neurons to model physiological events in vivo (Rose et al. 2013).

Various studies have used electrophysiology to assess the development of neurotransmitter release competence through development. In many cases, the parameter measured has been spontaneous, TTx‐sensitive EPSCs or IPSCs. However, spontaneous EPSCs (sEPSCs) and spontaneous IPSCs (sIPSCs) are a combination of both action potential‐independent and ‐dependent activity; therefore, with the exception of specific parameters (e.g., the frequency of TTx‐sensitive component), it is challenging to determine whether evoked SV exocytosis is altered purely via this measure. Nevertheless, in these studies, motoneurons in rat spinal cord preparations displayed an increase in both sEPSC and sIPSC frequency from E17–18 to P1–3 (Gao et al. 1998). In contrast, CA1–CA3 synapses within hippocampal slices displayed no obvious change in sEPSC frequency but a significant increase in amplitude from P9 to 3 months (Hsia et al. 1998). In primary cultures of medium spiny neurons from murine basal ganglia, which are mostly GABAergic, a similar increase in the amplitude of TTx‐sensitive sIPSCs was observed from DIV 7–8 to DIV 12–14 (Arama et al. 2015). However, as discussed above, dissection of canonical evoked and spontaneous release requires further experimentation, with changes in sE/IPSC amplitude most likely driven by alterations in postsynaptic receptor numbers.

One electrophysiological parameter that provides a unique insight into a specific aspect of presynaptic development: quantal size (*q*). This parameter refers to the amplitude of a single SV fusion event, or in other words, the amount of neurotransmitter packaged in one SV. However, when studies examining quantal size have been performed, the outcome appears complex. For example, the mean mEPSC amplitude at the calyx of Held was not significantly different between P7 and P14 (Iwasaki and Takahashi 2001). Similarly, at the cerebellar parallel fibre‐Purkinje neuron synapse, *q* was again comparable between P8–10 and P21–24 (Baur et al. 2015). However, when the same experiment was performed in layer 5 pyramidal neurons, a threefold increase in quantal size was observed within the same timeframe (Bornschein et al. 2019). Finally, at the basket cell‐Purkinje cell synapse, there was a decrease in *q* with increasing maturity along a similar time frame (Chen et al. 2024). In summary, there is no obvious convergence across different synapses in the developing brain with regard to the extent of neurotransmitter loading into SVs. This may reflect the individual requirements of specific synapses and circuits during synaptogenesis; however, a more detailed interrogation of this important process for synaptic physiology is required before a definitive conclusion can be reached.

In vivo, spontaneous network activity (SNA), which refers to a phenomenon of highly synchronous activity (‘waves’) in a circuit, is commonly observed during development in various brain regions including the hippocampus and neocortex. With increasing maturity, this phenomenon tapers off around the second postnatal week in rodents (Blankenship and Feller 2010; Kersbergen and Bergles 2024; Luhmann et al. 2016; Pires et al. 2021). While its initiation is speculated to be driven by spontaneous SV exocytosis, its propagation is likely to be mediated by evoked SV exocytosis of neurotransmitters with an additional contribution from gap junctions (Martini et al. 2021; Opitz et al. 2002; Pires et al. 2021). Manipulating SNA through genetic or pharmacological methods showed topological disruptions in various systems, such as defective segregation of the visual pathway, as reviewed previously (Martini et al. 2021; Wu, Kourdougli, and Portera‐Cailliau 2024). The molecular mechanism behind circuit refinement by SNA remains to be investigated, which would require a more thorough understanding of presynaptic neurotransmission in developing neurons.

## 4 SV Endocytosis

As described above, SV fusion with the plasma membrane releases neurotransmitter either spontaneously or in an evoked manner. Regardless of the mode of SV fusion, SV endocytosis is an essential step to regenerate SVs to replenish SV pools and maintain plasma membrane tension (Denker et al. 2011; Gan and Watanabe 2018). Surprisingly, relatively little is known regarding when and how SV endocytosis is recruited to the nascent presynapse.

Clues to the mode of endocytosis that is activated early in development could come from growth cones, since they display membrane turnover and appear to have discrete constitutive and evoked pathways (Cheng and Reese 1987; Diefenbach et al. 1999; Nozumi and Igarashi 2018). For example, using FM1‐43 to mark membrane retrieval in chick ciliary growth cones, a clamped depolarisation with high KCl resulted in internalisation of dye into endosomal structures. These endosomes could subsequently be stimulated to fuse and release dye on further KCl challenge (Diefenbach et al. 1999). Intriguingly, the same stimulus did not evoke dye release from endosomes formed via constitutive uptake, suggesting mechanistic segregation of these two recycling modes. More recent studies revealed the mechanism underpinning this basal membrane turnover, using uptake of a related dye, FM4‐64, at growth cones of cultured hippocampal neurons (Bonanomi et al. 2008). Pharmacological antagonism of actin polymerisation, phosphatidylinositol 3‐kinase activity and cholesterol function all inhibited basal FM4‐64 accumulation, but not knock‐down of clathrin heavy chain, suggesting this bulk uptake mode was similar to macropinocytosis. This was supported by molecular interventions that interfered with Rac1 function, a key regulator of this pathway (Bonanomi et al. 2008). Parallel studies examining the uptake of large dextran in chick dorsal root ganglion neurons supported the presence of a macropinocytosis pathway, with uptake inhibited by antagonists selective for membrane ruffling (Kabayama et al. 2009, 2011). It is unlikely that macropinocytosis is the early SV endocytosis mode in developing central nerve terminals; however, since (1) it is not activity‐dependent and (2) it does not select SV cargo (such as synaptophysin).

Aside from the endosomal structures formed in the growth cone, there are molecular similarities between SV endocytosis and growth cone repulsion. Growth cone repulsion is mediated by calcium influx, which leads to asymmetric endocytosis of one side of the growth cone in response to local guidance cues (Tojima et al. 2010). Experiments using total internal reflection fluorescence microscopy revealed that the recruitment of fluorescent‐tagged clathrin and dynamin‐1 was triggered in response to these repulsive guidance cues. Furthermore, growth cone repulsion was arrested using a series of pharmacological and molecular tools to inhibit either clathrin or dynamin‐1 function (Tojima et al. 2010). Furthermore, the recruitment of both clathrin and dynamin‐1 was blocked by inhibition of the calcium and calmodulin‐dependent protein phosphatase CaN, which activates dephosphins to trigger ADBE (Cousin and Robinson 2001; Tojima et al. 2010).

On the other hand, the requirement for CaN in endocytosis during presynaptic development is more nuanced. For example, SV endocytosis in both cultured cerebellar granule neurons and forebrain synaptosomes displayed limited sensitivity to the CaN antagonist cyclosporin‐A early in development (Smillie et al. 2005). In these studies, endocytosis was visualised via the activity‐dependent uptake and release of the dye FM2‐10, with cyclosporin‐A having little impact on cerebellar neurons at DIV 10, but robustly reducing SV retrieval at > DIV 30. Similarly, CaN‐dependence of SV endocytosis increased with age in forebrain synaptosomes from 2‐week‐old to 8‐month‐old rats, confirming the requirement for this phosphatase in mature, but not in immature nerve terminals (Smillie et al. 2005). On the contrary, SV endocytosis at the Calyx of Held synapse displayed reduced sensitivity to calmodulin and CaN antagonists from P7–9 to P13–14, when SV retrieval was monitored using membrane capacitance as a surrogate for surface area (Yamashita et al. 2010). The Wu group approached the same question with a genetic strategy, by generating CaN_A_ɑ knockout mice (Wu et al. 2014). The authors tested membrane capacitance at the Calyx of Held at P7‐10 and P13‐14, which marks before and after hearing onset, concomitant with tighter calcium coupling to SV exocytosis. In contrast to the previous study, the ablation of CaN_A_ɑ expression resulted in similar defects in SV endocytosis at both time points (Wu et al. 2014). Therefore, it may be that at small typical nerve terminals, CaN activity is dispensable for SV endocytosis during development, whereas in larger atypical boutons it is required. This may reflect the differences in calcium channel and microdomain organisation discussed above.

The study of SV endocytosis in typical small central terminals has been revolutionised with the advent of genetically encoded pHluorin reporters. These probes monitor the retrieval of SV cargo that they are fused to via quenching of their fluorescence on SV acidification subsequent to their retrieval. Therefore, information on SV cargo retrieval can be accessed at individual presynapses, if one assumes that the rate of SV acidification is constant. A recent study in cultured hippocampal neurons examined how quenching of the action potential‐evoked synaptophysin‐pHluorin signal altered through development. This study suggested that in nerve terminals that were competent to fuse SVs, there was no significant change in the kinetics of SV retrieval during neuronal maturation (Kim et al. 2024). This finding contrasts with studies in organotypic hippocampal slice cultures, which revealed a sevenfold acceleration in the rate of synaptophysin‐pHluorin quenching between DIV 5–7 to DIV 14–28 (Rose et al. 2013). Intriguingly, this developmental‐dependent acceleration, like the increased SV mobility, was not apparent in dissociated hippocampal cultures (Rose et al. 2013).

As stated in the introduction, various modes of endocytosis are proposed to coexist at the presynapse, namely CME, ADBE and UFE. Therefore, there is potential for granularity in selective repression or recruitment of individual modes to sculpt presynaptic function. The role of clathrin in SV cargo retrieval has been widely debated at mature nerve terminals in cultured neurons. For example, shRNA‐mediated knock‐down of its heavy or light chain has been shown to have either little impact (Kononenko et al. 2014; López‐Hernández et al. 2022; Soykan et al. 2016) or retard the retrieval of various pHluorin reporters (López‐Hernández et al. 2022; Lopez‐Murcia et al. 2014; Nicholson‐Fish et al. 2015; Wei et al. 2024). However, in immature presynapses, knock‐down of clathrin heavy chain accelerated the retrieval of synaptophysin‐pHluorin (Kim et al. 2024). Therefore, the role of clathrin, and potentially CME, is still unclear across the developmental spectrum. In contrast, endosome formation from the plasma membrane via either ADBE or UFE is universally agreed to be clathrin‐independent (Nicholson‐Fish et al. 2015; Watanabe et al. 2014). To determine when and if ADBE was recruited to nerve terminals during development, primary hippocampal cultures were challenged with high‐frequency action potential trains in the presence of the fluid‐phase marker tetramethylrhodamine (TMR)‐dextran, which is more likely to be internalised via ADBE rather than CME due to size exclusion (Clayton et al. 2008; Kim et al. 2024). Intriguingly, TMR‐dextran uptake was only apparent from DIV 14 onwards, a finding corroborated by an electron microscopy assay using the discrete fluid‐phase marker horseradish peroxidase (Kim et al. 2024). This late recruitment of ADBE was in contrast to the early adoption of activity‐dependent calcium influx, SV fusion and SV cargo retrieval, suggesting its activation may be repressed in early development.

A role for an ADBE‐like membrane retrieval pathway has been reported during remodelling of neuromuscular synapses in *Caenorhabditis elegans*. GABAergic presynapses in dorsal D motoneurons in larval *C. elegans* that innervate ventral muscle cells are eliminated as part of the rewiring process (Mulcahy et al. 2022). This elimination appears to require many of the key molecules required for ADBE, including CaN, dynamin, syndapin, the Arp2/3 complex and Rab‐11 (Cuentas‐Condori et al. 2023). The ADBE‐like pathway and presynaptic removal were triggered by activation of the epithelial sodium channel UNC8, with its depletion greatly retarding the appearance of endosomal‐like structures during remodelling (Cuentas‐Condori et al. 2023). In summary, there appear to be multiple forms of bulk membrane uptake at both central neuron growth cones and peripheral synapses in *C. elegans* larvae, with potentially overlapping mechanisms to those seen in mature neurons. Such findings imply that the molecular machinery for ADBE may be present in immature presynapses; however, the triggering mechanism may not be sufficient (such as inadequate calcium influx or SV exocytosis).

The final stage of SV recycling after endocytosis is the replenishment of SV pools. The nomenclature of these SV populations is not uniform, but typically they can be subdivided into SVs that are either accessible (recycling pool) or inaccessible (resting pool) to neuronal activity (Rizzoli and Betz 2005). The recycling pool can be subdivided into the readily releasable pool (RRP), which as the name suggests, are the SVs that fuse first on action potential invasion (Rosenmund and Stevens 1996) and the reserve pool (which repopulate the RRP). There are mixed opinions on whether the replenishment of SV pools differs between immature and mature neurons. Paired‐pulse or repeated stimulation protocols revealed that short‐term depression (typically viewed as depletion of available SVs) attenuates over development, suggesting that there is faster replenishment of SV pools in mature neurons (Bolshakov and Siegelbaum 1995; Cheetham and Fox 2010; Crins et al. 2011; Frick et al. 2007; Iwasaki and Takahashi 2001; Taschenberger and Von Gersdorff 2000). This suggests that SV endocytosis, recycling or both is upregulated with synaptic maturity. However, there is evidence that SV endocytosis is not significantly different across different ages. The slope of the cumulative EPSC amplitude plot under multiple stimulations, which is used as an indicator of presynaptic endocytosis rate in electrophysiology, was comparable between the first and the third postnatal week in parallel fibre‐Purkinje cell cerebellar synapses (Baur et al. 2015). The same experiment in layer 5 pyramidal neurons showed no significant difference in the replenishment rate of the RRP from P8–10 to P21–24 (Bornschein et al. 2020; Bornschein and Schmidt 2019). As an alternative explanation, they suggested that a finite ‘replenishing pool’ exists between the reserve pool and RRP, and that this pool only manifests itself at mature synapses (Bornschein et al. 2020; Bornschein and Schmidt 2019). Therefore, the balance of the current literature suggests that SV pool replenishment becomes more efficient with synaptic maturity.

## 5 Further Studies

The current state of knowledge regarding SV recycling mechanisms during presynaptic development is relatively sparse when compared to the abundance of information from studies in mature nerve terminals. From what is known, it appears that in immature neurons, calcium influx is less tightly coupled to SV fusion, the dominant neurotransmitter release mechanism is spontaneous exocytosis and while compensatory SV endocytosis occurs, there is a lack of clarity regarding the mechanism. This is in contrast to mature neurons, where SVs are more tightly coupled to activity‐dependent calcium influx, with the majority of neurotransmission synchronised to neuronal activity, and endosomal pathways (UFE/ADBE) appear to be dominant. In this final section, we highlight a number of aspects of SV recycling in immature nerve terminals that require further investigation and greater molecular and physiological insight.

### 5.1 Mechanism of Tightening of Calcium Coupling

The first obvious area for further investigation is the molecular mechanism behind the increase in coupling of calcium influx to SV fusion during maturation of the presynapse. In this regard, the filament‐forming GTPase septin‐5 is a lead candidate in coordinating this process. In the calyx of Held, it was suggested to act as a physical barrier between SVs and VGCCs at immature synapses. Septin‐5 filaments are absent in mature calyces, and mature calyces from septin‐5 knockout mice display an array of morphological and physiological similarities to immature calyces, including sensitivity to EGTA (Yang et al. 2010). The mechanism underpinning the elimination of septin‐5 filaments from the mature presynapse has still to be determined. This is of critical importance, since these filaments appear to be critical in the transition to establishing a high fidelity of stimulus–secretion coupling.

Another possible mechanism underpinning tighter calcium coupling is the progressive assembly of the presynaptic active zone. Although not covered in this review, the delivery and maturation of the active zone machinery are key initial steps in the establishment of the presynapse (Bolz and Haucke 2024; Gundelfinger et al. 2016). A key component of this is the delivery and tethering of VGCCs (Chin and Kaeser 2024; Held et al. 2020), with the latter requiring interactions with a series of cell adhesion molecules, such as the neurexins and laminins (Chen et al. 2011; Luo et al. 2020; Nishimune et al. 2004). Considering this globally, it is likely that the tighter coupling of calcium influx to SV fusion during presynaptic maturation is an integration of a number of interdependent processes including those addressed above.

One final factor in the efficiency of calcium coupling is [Ca^2+^]_i_ buffering. Inefficient buffering would result in further diffusion of calcium outside the microdomain, potentially triggering spontaneous/asynchronous SV fusion events mediated via high‐affinity calcium sensors such as Doc2 (Wu, Kusick, et al. 2024; Yao, Gaffaney, et al. 2011). The major [Ca^2+^]_i_ sink in nerve terminals is mitochondria, which limit action potential‐evoked [Ca^2+^]_i_ increases in nerve terminals (Devine and Kittler 2018). Other key regulators are calcium pumps on both the plasma membrane and endoplasmic reticulum, which sequester [Ca^2+^]_i_ (Billups and Forsythe 2002; Chanaday and Kavalali 2022; Wan et al. 2012). Calcium clearance in nerve terminals in primary neuronal culture appears to be optimal by 3 DIV (Kim et al. 2024); however, methods to monitor bulk [Ca^2+^]_i_ increases will not detect alterations below the resolution of light microscopy. Therefore, whether calcium buffering mechanisms perform subtle roles to control SV recycling through development is still open to question.

### 5.2 Mechanism of Developmental Switch From Spontaneous to Evoked Neurotransmitter Release

A related question to address is a determination of the molecular mechanism that underpins the mode switch of SV exocytosis from spontaneous to evoked during presynaptic maturation. As stated above, inhibition of voltage‐gated sodium channels in immature neurons using TTx had little effect on presynaptic activity (Andreae et al. 2012; Groc et al. 2002; Hsia et al. 1998). Therefore, it is essential to determine the link between neuronal activity and the coupling of VGCCs to SV fusion through development. Intriguingly, disruption of microtubule filaments in mature cholinergic nerve terminals via a variety of interventions resulted in increased spontaneous neurotransmitter release (Velasco et al. 2023). Similarly, knockout of β‐actin resulted in a dramatic increase in spontaneous SV fusion events (Wu et al. 2025) similar to previous interventions that depolymerise actin microfilaments (Morales et al. 2000). Therefore, both microtubules and microfilaments appear to repress spontaneous SV fusion, suggesting their ingress into the maturing presynapse may dictate a transition from spontaneous to evoked neurotransmitter release.

An equally plausible and complementary explanation for the developmental switch in the mode of SV fusion is a change in the composition of SVs. For example, in mature nerve terminals, a series of v‐SNARE proteins including Vti1a, VAMP4 and VAMP7 all appear to bias neurotransmitter release towards spontaneous SV fusion when overexpressed in primary neuronal cultures (Bal et al. 2013; Lin et al. 2020; Ramirez et al. 2012). Intriguingly, some of these proteins are also selectively accumulated by specific endocytosis modes (Nicholson‐Fish et al. 2015). Therefore, the composition of SVs through development, and the endocytosis mode that generates them, may be critical in determining which SV fusion mode predominates.

In this regard, one key SV protein that has established roles in the control of both SV fusion modes and SV endocytosis is the calcium sensor Syt1. Syt1 is essential for optimal SV endocytosis, although its mechanism is still debated (Bolz et al. 2023; Yao, Kwon, et al. 2011). However, it is established that Syt1 plays a key role in the repression of spontaneous SV fusion and the synchronisation of neurotransmitter release to calcium influx. Specifically, mature Syt1 knockout neurons display an upregulation of spontaneous SV fusion events, in addition to ablation of synchronous neurotransmitter release (Geppert et al. 1994). Interestingly, when the frequency of spontaneous mEPSC events was compared between primary cultures of wild‐type and Syt1 knockout neurons, there was no difference between the genotypes at DIV11–12, whereas after this timepoint the frequency decreased in wild‐type but increased in knockout (Bouazza‐Arostegui et al. 2022). This is consistent with a model in which the appearance of Syt1 on SVs represses spontaneous fusion while synchronising neurotransmitter release. It will be essential to determine the molecular inventory of SVs at specific developmental timepoints to confirm or disprove these hypotheses.

### 5.3 Endocytosis Modes Through Development

How SV endocytosis is recruited and how it sustains neuronal activity at different stages in presynaptic development is another key question. It is likely that CME is a basal mechanism that enables membrane homeostasis and axon guidance from the early stage of presynaptic differentiation, as demonstrated in the growth cone (Tojima et al. 2010). If CME is operational at this early stage, it would ensure that SVs generated from the plasma membrane would have the correct molecular composition to be competent for neurotransmitter uptake and release. Basal fluid‐phase uptake mechanisms are also present at the nascent presynapse, which can contribute membrane towards future SV fusion events (Diefenbach et al. 1999; Bonanomi et al. 2008). However, these pathways are not coupled to neuronal activity, unlike UFE and ADBE. In this context, the recent finding that ADBE was only recruited for use from the second week in primary hippocampal culture was surprising, considering that SV fusion events were occurring significantly earlier. This recruitment of ADBE coincides with the mode switch of SV exocytosis from spontaneous to evoked (Andreae et al. 2012; Kim et al. 2024), potentially explaining its appearance at this specific developmental time point. It is possible that the molecular machinery for ADBE is present at the presynapse earlier in development; however, it is only triggered by the sudden increase in plasma membrane surface area generated by synchronous SV exocytosis (Ogunmowo et al. 2023).

Alternatively, ADBE may be occurring at earlier developmental timepoints, but the diameter of the plasma membrane invagination is smaller, which will exclude traditional fluid‐phase markers such as TMR‐dextran or horseradish peroxidase. This is supported by studies that report the evoked uptake of FM dyes and Syt1 antibodies before DIV14 in culture (Diefenbach et al. 1999; Kraszewski et al. 1995; Coco et al. 2002). Finally, ADBE is coupled to neuronal activity by delocalised calcium increases that activate CaN (Morton et al. 2015). Therefore, the triggering of ADBE may also be dependent on the maturation of VGCC topography at the active zone. Further studies will require a careful disentangling of all of these potential overlapping scenarios before a more definitive conclusion can be made regarding the sequential activation of endocytosis modes during presynaptic maturation.

Finally, the delayed recruitment of ADBE in comparison to other SV endocytosis modes has potential implications for human disease. This is because when the triggering of ADBE was examined in neurons that modelled the neurodevelopmental disorder Fragile X Syndrome, there was an exacerbation of this delay (Kim et al. 2024). Importantly, a detailed screen of SV recycling in five independent rodent models of Autism Spectrum Disorder revealed that the only deficit was a conserved reduction of ADBE (Bonnycastle et al. 2025). Therefore, a delay in the recruitment of ADBE could be a conserved mechanism that contributes to either the pathology of neurodevelopmental disorders or a compensatory mechanism to alleviate dysfunction. It will be imperative for future studies to delineate between these opposing scenarios, since the conclusion will have the potential for a novel therapeutic avenue to pursue.

## 6 Conclusion

As highlighted in this article, the study of SV recycling mechanisms in the developing presynapse has lagged behind those in mature nerve terminals. However, studying the distinct neurotransmission mechanisms of developing presynapses will be critical in understanding how presynaptic development sculpts eventual circuit and brain function. Furthermore, it will provide essential evidence from which to focus research into human physiology and pathology, especially in neurodevelopmental disorders.

## Author Contributions

**Nawon Kim:** writing – original draft, writing – review and editing. **Michael A. Cousin:** supervision, writing – review and editing.

## Conflicts of Interest

Michael A. Cousin is a handling editor for the *Journal of Neurochemistry*.

## Data Availability

Data sharing is not applicable to this article as no datasets were generated or analysed during the current study.
